# Balancing Outcome vs. Urgency in Modern Liver Transplantation

**DOI:** 10.3389/fsurg.2022.853727

**Published:** 2022-03-04

**Authors:** Peter T. Dancs, Fuat H. Saner, Tamas Benkö, Ernesto P. Molmenti, Matthias Büchter, Andreas Paul, Dieter P. Hoyer

**Affiliations:** ^1^General, Visceral and Transplantation Surgery, University Hospital Essen, Essen, Germany; ^2^Department of Surgery, Northwell Health, Donald and Barbara Zucker School of Medicine at Hofstra/Northwell, Hofstra University, Hempstead, NY, United States; ^3^Gastroenterology and Hepatology, University Hospital Essen, Essen, Germany

**Keywords:** outcome, waiting list, urgency, organ allocation, liver transplantation

## Abstract

**Background:**

Current allocation mechanisms for liver transplantation (LT) overemphasize emergency, leading to poorer longtime outcomes. The utility was introduced to recognized outcomes in allocation. Recently, Molinari proposed a predictive outcome model based on recipient data.

**Aims:**

The aims of this study were to validate this model and to combine it with the utility to emphasize outcome in allocation.

**Methods:**

We retrospectively analyzed 734 patients who were transplanted between January 2010 and December 2019. Points were assigned as in Molinari's model and the score sum was correlated with observed 90-day mortality. The utility was calculated as the product of 1-year survival times 3-month mortality on the waiting list. The weighting of different compounds was introduced, and utility curves were calculated. Model for End-Stage Liver Disease (MELD) scores according to maximal utility were determined.

**Results:**

In total, 120 patients (16.3%) had died within 90 days after LT. Higher MELD score, obesity, and hemodialysis prior to LT were confirmed risk factors. Overall survival was 83.8 and 77.4% after 90 days and 12 months, respectively. General utility culminated at MELD scores >35 in the overall population. Emphasizing the outcome shifted the maximal utility to lower MELD scores depending on Molinari scores.

**Conclusions:**

Emphasizing outcome, at least in certain recipient risk categories, might improve the longtime outcomes and might be integrated into allocation models.

## Introduction

The ongoing scarcity of deceased donor organs for liver transplantation (LT) has led to the development of various allocation systems in the past decades (1–5). Based on the principles of emergency, outcome, and fairness, the current system advocates Model for End-Stage Liver Disease (MELD) scores to prioritize the sickest patients in need of immediate transplantation. Such emergency-driven allocation, based on the “sickest first” principle, shifts waiting list mortality (6) to the post-transplant period, resulting in poorer outcomes (7, 8). Donor risk factors can further negatively influence such outcomes, as encountered in the EUROTRANSPLANT (ET) region (9–12).

Utility represents a potential model to reconcile the frequently diverging concepts of emergency-driven transplantation and outcome optimization (13). Utility (U) of LT is defined as the product of outcome (O = 1-year survival with LT) times emergency (E = 3-month mortality without LT), or U = O × E. It entails the transplantation of those candidates where the allocation of organs will be optimized. An appealing model for such allocation is the predictive outcome model recently published by Molinari et al. (14), which is solely based on recipient parameters available prior to organ acceptance. The aim of the present study was to: (1) validate this new predictive outcome model in a high-volume ET liver transplant center, and (2) combine this model and utility to determine the optimal weighting of emergency and outcome.

## Methods

### Study Population

We performed a retrospective single-center analysis concerning all consecutive LTs from January 2010 to December 2019 at the University Hospital Essen, Germany. Recipients <18 years of age, retransplants, recipients of multiorgan grafts, and living donor allografts were excluded from the analysis. All livers were donated by brain dead heart-beating donors. In accordance with the local and ET guidelines, all transplantations were carried out between donors and recipients of compatible ABO blood type. All data were collected from our own databases and the EUROTRANSPLANT International Foundation. The study was approved by the local Ethics Committee.

### Surgery and Immunosuppression

Procurements were carried out in a standard fashion as defined by ET (15). Transplants were performed with inferior vena cava replacement and end-to-end-anastomoses of the hepatic artery, portal vein, and bile duct. Bypass techniques are not used. The regimen of immunosuppression was standardized with intravenous corticosteroids at the time of transplantation with subsequent post-operative tapering, calcineurin inhibitors (tacrolimus trough level 6–8 ng/ml), and mycophenolate mofetil (0.5–1 g, twice daily). All patients were treated and observed post-operatively at a liver transplant intensive care unit (ICU).

### Molinari Score Model

A point-based scoring model based on cutting-edge statistics (artificial neural networks, classification tree analysis) was recently developed by Molinari et al. (14). This model is unique in its ability to predict the post-operative outcome after LT based on recipient characteristics. Variables of the score are based on age, MELD score, body mass index (BMI), presence of Type 1 or 2 Diabetes, and the need for pretransplant dialysis. All recipient parameters in our study population were reassessed by logistic regression analysis, with 90-day mortality as our end point since the original artificial neural network was not available. Points were assigned for parameters with statistical significance in accordance with the weighing of the original Molinari model. Cut-offs were selected on recalculated receiver operator characteristic (ROC) analyses and Youden indices in the present cohort. Categorical factors were provided one point. The maximum number of points that each patient could score was limited to 5 (due to few patients with higher scores). The score sum was correlated with the observed 90-day mortality. The point-based observed 90-day mortality was correlated with the predicted 90-day mortality (from logistic regression models).

### Balance of Emergency and Outcome

The concept of balancing emergency and outcome in LT has been proposed previously (16–18). “Utility” refers to the allocation of organs to recipients who will make the best use of them and from a methodological point of view. It can be calculated as the product of outcome and emergency:


(1) $$\begin{array}{r} Utility\;\left(U\right)=Outcome\;\left(O\right)\;\times \;Emergency\;\left(E\right) \end{array}$$

The outcome is denoted by 1-year survival as a function of the MELD score.

An emergency is represented by the 3-month mortality on the waiting list as a function of the MELD score.

This relationship can be further adapted by the so-called Cobb-Douglas function that provides the opportunity of weighting emergency or outcome:


(2) $$\begin{array}{r} U_{\text{w}}=O^{\text{a}}\;\times \;E^{\text{b}} \end{array}$$

where U_w_ = weighted utility and

a + b = 1 [representing the chosen weighing of emergency or outcome (e.g., weighing of 4/1 results in: a = 0.8 and b = 0.2)].

O = 1-year survival after LT determined for every single MELD score and

E = calculated 3-month mortality for every single MELD score.

Model for End-Stage Liver Disease-dependent survival after LT (extracted from our database) is then plotted against the MELD-dependent 3-month mortality (obtained from publicly available data) and a utility curve was calculated point by point.

Present allocation mechanisms, driven by the MELD scores, solely emphasize emergency irrespective of the outcomes. Since the inclusion of utility in allocation scores entails a subjective component, we provide two different weighting options. Considering the dominance of the logarithmic equation of the MELD-dependent emergency in the Cobb-Douglas function, we shifted weight toward an outcome, to achieve a better balance of both contributing parameters. Sensitivity analysis (effect sizes, data not shown) demonstrates the least bias by one or both factors when outcome contributes four or nine times more to overall utility than an emergency (a and b would be 0.8 and 0.2, or 0.9 and 0.1, respectively).

### Utility in Different Molinari Score Risk Categories

We applied the concept of balancing outcome and emergency in different categories of the Molinari score to account for 90-day recipient mortality risk after LT. MELD-associated points of the Molinari score were subtracted from the sum for each patient since MELD was already the survival-defining parameter. Three different Molinari score categories were found in our population: 0, 1, and 2 points. We subsequently extracted for each of these categories the 1-year survival data based on MELD scores and utilized them as “Outcome”. The “Emergency” based on MELD scores was represented by the 3-month waiting list mortality (19). Utility and weighted utility [4/1 (U_w_ = O^0.8^ × E^0.2^) and 9/1 (U_w_ = O^0.9^ × E^0.1^)] were calculated for each scoring category.

### Statistics

Continuous variables are presented as means or median and SD and range as appropriate. Categorical data are depicted by frequency and percentages. ANOVA, χ^2^, and Kruskal–Wallis tests were used for group comparisons. Survival analysis was performed using the Kaplan–Meier method. Cox regression analysis was performed to identify independent risk factors for overall survival. Logistic regression was utilized to assess the effect of independent risk factors on 90-day mortality. ROC analyses were performed and Youden indices were calculated to identify optimal cut-off values. All statistical analyses were performed using SPSS Statistics for Windows, Version 24 (IBM Corporation, Armonk, NY, USA) and Excel (Microsoft Corporation, Redmond, WA, USA). A *p* of < 0.05 was considered statistically significant, and two-tailed tests were used for all statistical analyses.

## Results

### Study Population

In total, 734 consecutive patients underwent primary LT between January 2010 and December 2019. Patient characteristics are shown in Table 1. In total, 120 recipients (16.3%) died within 90 days after LT. The 90-day mortality risk factors included higher MELD scores, obesity, and hemodialysis prior to transplantation. Recipients who died during this time period also tended to be older.

**Table 1:** Demographic and clinical characteristics of patients who deceased within 90 days, patients who survived beyond 90 days after liver transplantation (LT) and the entire study population, respectively.

	**Patients who died within 90 days**	**Patients who survived beyond 90 days**	**Entire cohort**	
**Characteristics**	***n* = 120**	***n* = 614**	***n* = 734**	***P***
Age, years, mean (SD)	53.9 (11.5)	51.8 (11.0)	52.1 (11.1)	0.0580
**Sex**, ***n*** **(%)**				
Female	38 (31.7)	209 (34.0)	247 (33.7)	0.6149
Male	82 (68.3)	405 (66.0)	487 (66.3)	
MELD score, mean (SD)	22.9 (10.7)	16.9 (8.5)	17.8 (9.2)	<0.0001
**MELD score**, ***n*** **(%)**				
<15	34 (28.3)	296 (48.2)	330 (45.0)	
15–20	29 (24.2)	166 (27.0)	195 (26.6)	
21–25	12 (10.0)	66 (10.7)	78 (10.6)	
26–30	4 (3.3)	20 (3.3)	24 (3.3)	
>30	41 (34.2)	66 (10.7)	107 (14.6)	
Diabetes (type I or II), *n* (%)	27 (22.5)	129 (21.0)	156 (21.3)	0.7151
Need for dialysis before LT, *n* (%)	14 (11.7)	33 (5.4)	47 (6.4)	0.0100
BMI, kg/m^2^, mean (SD)	28.1 (5.4)	25.9 (4.8)	26.3 (4.9)	<0.0001
**BMI**, ***n*** **(%)**				
Underweight (<18.5)	3 (2.5)	17 (2.8)	20 (2.7)	
Normal weight (18.5–24.9)	37 (30.8)	269 (43.8)	306 (41.7)	
Overweight (25–29.9)	36 (30.0)	223 (36.3)	259 (35.3)	
Class I obesity (30–34.9)	30 (25.0)	73 (11.9)	103 (14.0)	
Class II obesity (35–39.9)	13 (10.8)	26 (4.2)	39 (5.3)	
Class III obesity (40–44.9)	1 (0.8)	6 (1.0)	7 (1.0)	
Super obesity (≥45)	0 (0.0)	0 (0.0)	0 (0.0)	
**Primary indication for LT**, ***n*** **(%)**				
Viral hepatitis	31 (25.8)	183 (29.8)	214 (29.2)	0.3813
NASH	15 (12.5)	58 (9.4)	73 (9.9)	0.3066
ALD	34 (28.3)	194 (31.6)	228 (31.1)	0.4799
AIH	7 (5.8)	27 (4.4)	34 (4.6)	0.4937
PBC	3 (2.5)	25 (4.1)	28 (3.8)	0.4110
PSC	7 (5.8)	45 (7.3)	52 (7.1)	0.5592
Toxic	6 (5.0)	13 (2.1)	19 (2.6)	0.0689
HCC	22 (18.3)	156 (23.8)	168 (22.9)	0.1941
Other	23 (19.2)	143 (23.3)	166 (22.6)	

*NASH, non-alcoholic steatohepatitis; ALD, alcoholic liver disease; AIH, autoimmune hepatitis; PBC, primary biliary cirrhosis; PSC, primary sclerosing cholangitis; HCC, hepatocellular carcinoma*.

### Patient Outcomes

**Figure 1:**
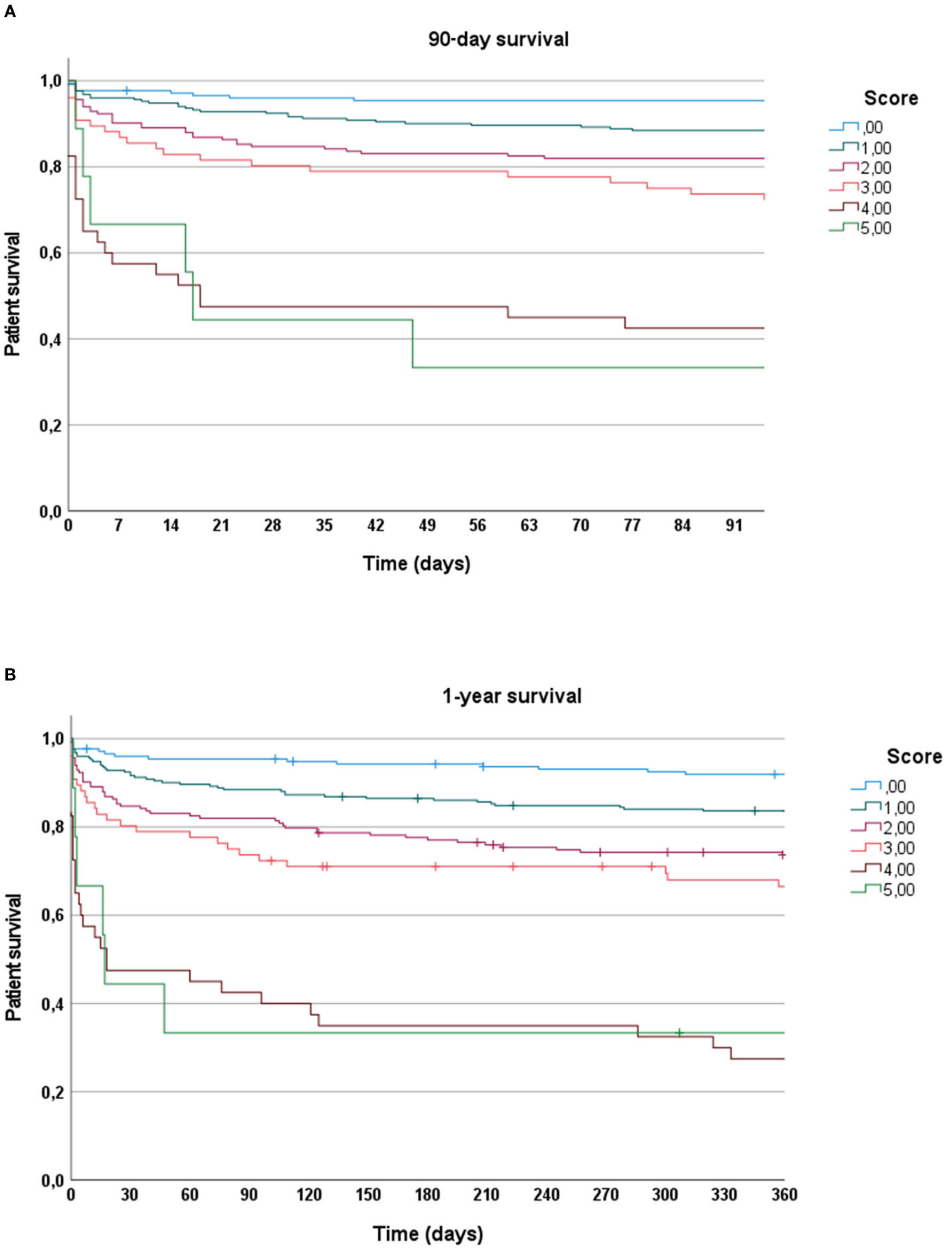
**(A)** Kaplan–Meier analysis depicting 90-day survival according to Molinari score. **(B)** Kaplan–Meier analysis depicting 1-year survival according to Molinari score.

Overall patient survival after 90 days and 12 months was 83.8 and 77.4%, respectively. Kaplan-Meier analysis of patient survival according to the Molinari Score is depicted in Figure 1. Statistical significance was observed between the groups (log-rank *p* <0.0001).

### Molinari Score

**Figure 2:**
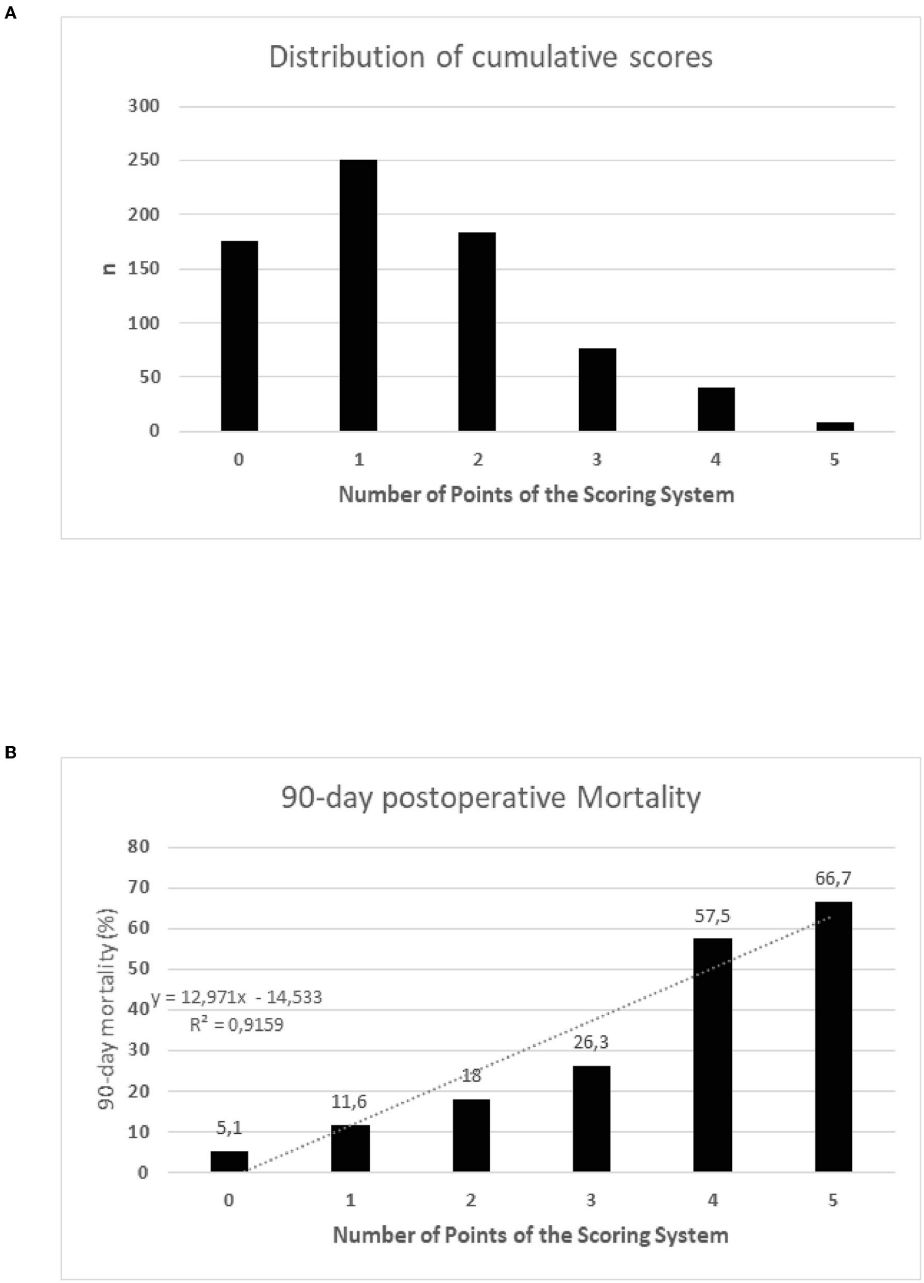
**(A)** Distribution of the cumulative Molinari scores. **(B)** Observed 90-day mortality stratified by the cumulative points of the scoring system. Mortality significantly differed between all groups (*p* = 0.001). Group comparisons demonstrated a significant increase of mortality based on cumulative scores (0 points vs. 1 point, *p* = 0.04; 1 point vs. 2 points, *p* = 0.06; 2 points vs. 3 points, *p* = 0.49; 3 points vs. 4 points, *p* = 0.01; 4 points vs. 5 points, *p* = 0.59).

The original Molinari score was applied to the present patient cohort and showed 382 patients (52%) with 0 points, 205 (27.9%) with 1 point, 57 (7.8%) with 2 points, 51 (6.9%) with 3 points, 35 (4.8%) with 4 points, and 4 (0.5%) with 5 points. The predictive power of this point-based system and the single parameters to predict 90-day mortality, evaluated by logistic regression and ROC analysis (data not shown), demonstrated only a limited performance quality with an area under the curve (AUC) of 0.58. Multivariable logistic regression analysis identified recipient age, labMELD score, and BMI as independent predictors of 90-day mortality. Cut-off values for labMELD and BMI were determined based on Youden indices, and points were given based on the Molinari scoring. The resulting point-based system and the distribution of points are presented in Table 2. Cumulative scores (Figure 2A) were capped at five points due to a limited number of patients having higher values. A 90-day mortality based on this adapted Molinari score is shown in Figure 2B. This adapted scoring system demonstrated a good predictive ability by ROC analysis (AUC of 0.74) and a Pearson correlation coefficient (*R*^2^) observed and predicted 90-day mortality of 0.97.

**Table 2:** Preoperative patient characteristics identified as independent predictors of 90-day mortality after liver transplantation (LT) and resulting scores.

**Patient characteristics**		**Points**	**Distribution (*n*/%)**
Age (years)	<55	0	384 (52.3)
	55–64	1	288 (39.2)
	65–70	2	56 (7.6)
	>70	3	6 (0.8)
Lab MELD score	<17.5	0	449 (61.2)
	17.5–30	1	178 (24.3)
	30–35	2	35 (4.8)
	>35	3	72 (9.8)
BMI (kg/m^2^)	<18.5	1	20 (2.7)
	18.5–30	0	565 (77)
	>30	1	149 (20.3)

*The distribution of the single factors is displayed additionally. BMI, body mass index*.

### Balance of Emergency and Outcome—All LT

**Figure 3:**
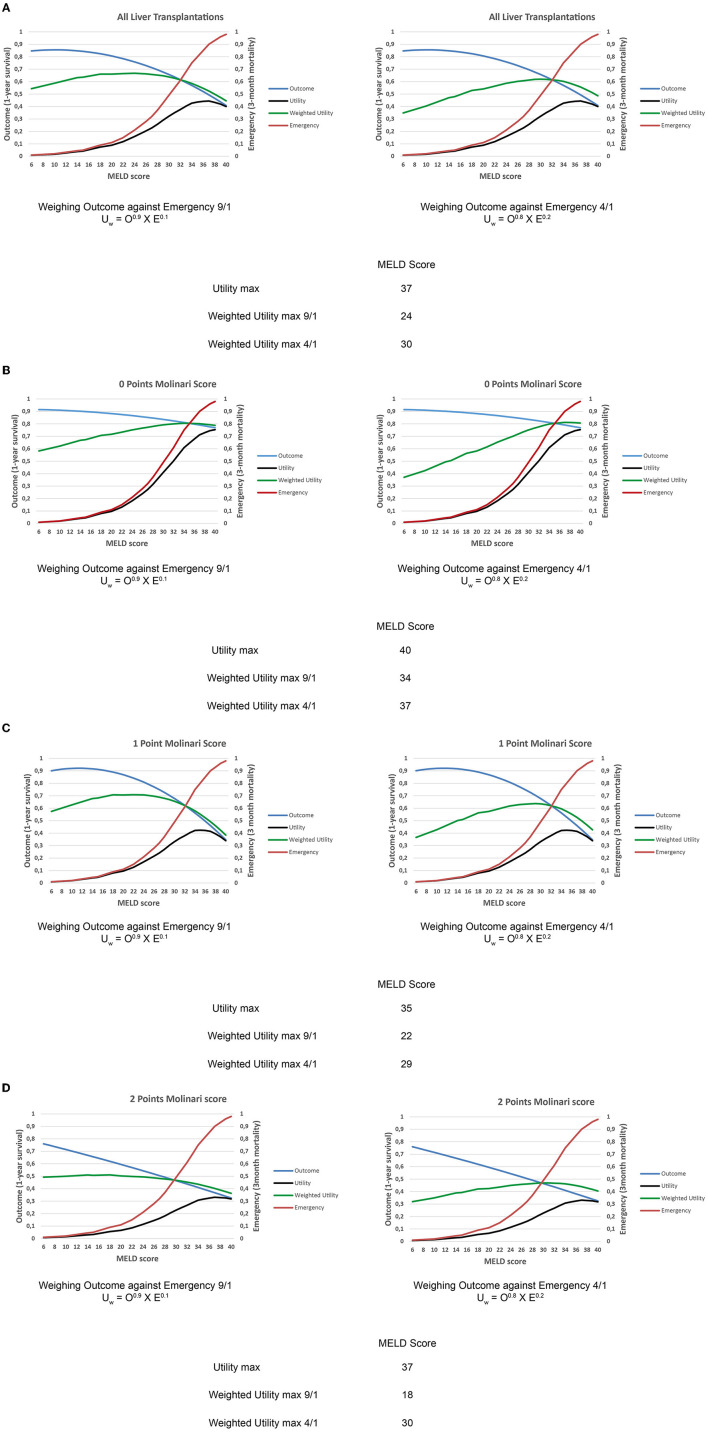
**(A)** Weighted utility curves for all liver LT patients based on outcome [1-year survival after liver transplantation (LT) determined for every single Model for End-Stage Liver Disease (MELD) score value] and emergency (calculated 3-month mortality without LT upon MELD score) with a weighing of the outcome against emergency 9:1 and 4:1, respectively. The peak cuspidal point of the weighted utility curve represents the MELD zone of the highest utility. **(B)** Weighted utility curves for low-risk recipients (0 Molinari points) based on outcome [1-year survival after liver transplantation (LT) determined for every single Model for End-Stage Liver Disease (MELD) score value] and emergency (calculated 3-month mortality without LT upon MELD score) with a weighing of the outcome against emergency 9:1 and 4:1, respectively. The peak cuspidal point of the weighted utility curve represents the MELD zone of the highest utility. **(C)** Weighted utility curves for medium-risk recipients (1 Molinari point) based on outcome [1-year survival after liver transplantation (LT) determined for every single Model for End-Stage Liver Disease (MELD) score value] and emergency (calculated 3-month mortality without LT upon MELD score) with a weighing of the outcome against emergency 9:1 and 4:1, respectively. The peak cuspidal point of the weighted utility curve represents the MELD zone of the highest utility. **(D)** Weighted utility curves for high-risk recipients (2 Molinari points) based on outcome [1-year survival after liver transplantation (LT) determined for every single Model for End-Stage Liver Disease (MELD) score value] and emergency (calculated 3-month mortality without LT upon MELD score) with a weighing of the outcome against emergency 9:1 and 4:1, respectively. The peak cuspidal point of the weighted utility curve represents the MELD zone of the highest utility.

Maximal utility with the equally weighted outcome and emergency (U = O × E) was observed at a MELD score of 37. Emphasizing on outcome weighing of 4/1 or 9/1 vs. emergency altered the shape of the utility curves and reduced the maximum value of utility to a MELD scores of 30 and 24, respectively. The cumulative utility overall MELD scores was greater for 9/1 than for 4/1 (13.79 vs. 12.68, respectively; Figure 3A).

### Balance of Emergency and Outcome—Recipient Risk Stratified

We applied the same type of analysis to various recipient risk categories as stratified by the Molinari scoring system (with subtraction of the MELD-based points). Accordingly, outcome data were different from the initial balance of emergency and outcome.

For recipients with 0 points, the maximum value for the general utility calculation was observed at a MELD score of 40. Weighted utility curves with a weighing of 4/1 and 9/1 demonstrated a maximum value of weighted utility at MELD scores of 37 and 34, respectively. The cumulative utility was greater for 9/1 than for 4/1 (17.34 vs. 15.78, respectively; Figure 3B).

Recipients with 1 Molinari point had a maximum value for utility at a MELD score of 35. Weighting utility at a 4/1 ratio led to a maximum value at a MELD score of 29, with a cumulative utility of 12.82. Weighting utility at a 9/1 ratio led to a maximum value at a MELD score of 22, with a cumulative utility of 14.06 (Figure 3C).

Recipients with 2 Molinari points showed a maximum general utility value at a MELD score of 37. In this group of patients with inferior outcomes, maximum values for weighting of 4/1 and 9/1 were observed at MELD scores of 30 and 18, respectively. The cumulative utility was slightly higher for 9/1 than 4/1 (10.56 vs. 9.96, respectively). The weighted curves for this group of recipients were flatter than for the other Molinari groups (Figure 3D).

## Discussion

In the present study we aimed to 1. validate the recently published model of Molinari et al. for the prediction of 90-day mortality after LT based solely on recipient parameters, 2. utilize this model to optimize the weighting balance between emergency and outcome considerations based on objective parameters. Since the application of the original model and scoring system provided only a moderate ability to predict 90-day mortality in our cohort, we adapted the scoring system based on multivariable logistic regression analysis and revised cut-offs for significant parameters. Our final model for 90-day mortality prediction included recipient age, BMI, and labMELD scores. The resulting point-based system in Molinari scoring style is presented in Table 2. Recipient diabetic status, although predictive of overall mortality by Cox regression analysis, did not achieve significance for 90-day mortality (data not shown). This is consistent with previous studies (20, 21) and with the observation that poorly controlled blood glucose levels are predominantly associated with cardiovascular and neurological events.

It has been frequently proposed that organ allocation should have a multi-faceted approach and not be based on a “sickest-first” (emergency) concept. In the modern era of organ transplantation, with a persistent severe shortage of available organs, post-transplant outcomes are of utmost relevance (22). Since the introduction of the MELD-based organ allocation in the ET area, emergency alone has been the sole driving force in organ allocation. Although this allocation mechanism was shown to perform well from a recipient benefit standpoint (23, 24), the data for these studies were based on historic samples with the underlying clinical bias of the best organs transplanted into the highest risk recipients. A different approach was previously described by Burton et al. (13), where emergency and outcome were balanced against each other to optimize transplantation. We applied this rationale in our study based on the model of recipient risk assessment developed by Molinari. Our results provided several interesting observations. First, the simple combination of outcome and emergency in LT recipients (without considering risk profiles) leads to the highest utility in high MELD recipients (labMELD of 37), consistent with the current sickest-first concept. Emergency (as depicted by MELD scores) greatly influences utility due to the steep rise in higher MELD patients, with only a moderate decline in outcomes. Rather than implying that only patients with MELD scores in the upper 30s should be transplanted, this illustrates that the utility curve is influenced primarily by emergency rather than outcome. From a mathematical perspective, an emergency is over-emphasized in this scenario. To account for this effect, although shifting weight on outcome seems reasonable, socio-ethical opinions still need to be considered.

*Outcome/emergency weighting options of 4/1 and 9/1* (chosen because they had the least influence of both combined factors on the resulting utility curves) demonstrated a shift of the highest utility to a MELD score of 30 or 24, respectively. The introduction of recipient risk profiles provided a distinct pattern of maximized utility. *In recipients with low-risk profiles*, the highest utility was observed again with higher MELD scores (37 or 34) (Figure 3B). This underlines the superb outcomes that can be achieved in such recipients, even in the setting of advanced liver disease. Moreover, the curve of the weighted utility demonstrated a consequent rise over MELD scores, justifying the sickest-first approach for these recipients. *In medium risk recipients* (*1 point*), the weighted utility peaked at 29 and 22 MELD points. This is a relevant shift compared to patients with low-risk profiles. Indeed, the round outline of the curves with a clear decline of the weighted utility curves in the high MELD area demonstrates distinct susceptibility to the MELD score. The decline in the upper MELD area shows that for recipients with moderate risk, outcomes are dependent on the overall condition of the candidate. Blunt application of the sickest-first principle in this cohort of patients seems unreasonable if the outcome is considered for allocation purposes. Finally, in the case of *higher risk recipients* (*2 points*), the weighted utility showed maximum values at 30 and 18 MELD points. Additionally, the curves of the weighted utility are flattened compared to the other graphs. This reflects the oppositional influence of the MELD score on outcome and emergency for these recipients. In this group, the weighting of outcome becomes more important (reflected by the prominent difference of maximal weighted utility MELD scores 18/30), as declining outcomes are observed in higher MELD recipients with greater risk factors. In this group of recipients, the “sickest-first” principle is also challenged by the observed results.

Overall, our data demonstrate the very strong influence of emergency in the present “sickest-first” allocation system. While such rationale is supported by healthy recipients with no MELD-independent risk factors, other recipients with such findings will probably benefit from earlier transplantation with lower MELD scores. The maximum utility value does not necessarily imply that beyond the described MELD score, transplantation is futile. It only defines the MELD area with optimized achievable outcomes when considering the risks and conditions of the recipient. Moreover, a lower cut-off MELD score at which relevant utility is achieved should not be drawn from the present data (not presented in the current manuscript).

Modifications of the current system have to be implemented to better allocate scarce organs to the various recipient risk categories. Allocation mechanisms in LT have evolved over time. Initially, transplant surgeons selected recipients by subjective rationality. Subsequently, potential recipients were listed, and wait time was utilized to add fairness to the allocation process. Thereafter, more elaborated concepts took shape. Patients were categorized based on disease severity, initially determined by the level of medical attention required and then according to the Child-Turcotte-Pugh Score. Ultimately, the emergency-driven MELD score became dominant and gained acceptance worldwide (2). Currently, the system is being re-evaluated (25), and both donor (26–28) and recipient (18) characteristics are being discussed. The Molinari scoring was developed by state-of-the-art statistical methods based solely on recipient variables available at the time of organ offer. Our present cohort corroborated its ability to predict short- and long-term outcomes (Figure 1).

An intriguing aspect of LT is the diseases, in which the labMELD score fails to adequately describe the severity of the condition and survival of the patient. These diseases include, e.g., hepatocellular carcinoma, primary sclerosing cholangitis, and polycystic liver disease. For these cases, a standard-exceptional MELD (seMELD) score was introduced to counteract this kind of disadvantage of the system in the case of these patients. In our study, we did not exclude these patients, however, in these cases, labMELD score was taken into consideration to calculate the Molinari score. The labMELD score, calculated from the de facto liver function, may serve this purpose more accurately.

Like any other study, our data have limitations. As a retrospective analysis, it provides less persuasive power than a prospective pragmatic trial. Another factor to consider is the monocentric design, which carries the risk of bias. However, our study displays some strengths. Every patient underwent very similar standard surgical procedures and was treated postoperatively at our liver transplant ICU under high-standard protocols, which provides an outstanding comparability of all recipients.

In conclusion, our data demonstrate the dominant impact of the MELD score in the present allocation model. An emphasis on outcome, at least for certain recipient MELD-independent risk categories, might improve the longtime outcomes and might be integrated into the allocation process. Even if the described system does not become the standard mode of organ allocation, it still represents a useful tool for transplant physicians when considering individual candidates. The ratio of outcome/emergency weighting remains a matter of debate.

## Data Availability Statement

The raw data supporting the conclusions of this article will be made available by the authors, without undue reservation.

## Ethics Statement

Ethical review and approval was not required for the study on human participants in accordance with the local legislation and institutional requirements. Written informed consent for participation was not required for this study in accordance with the national legislation and the institutional requirements.

## Author Contributions

PD and DH designed the study. PD, DH, and MB evaluated the data. DH, PD, and FS wrote the manuscript. EM, TB, and AP corrected the manuscript. All authors contributed to the article and approved the submitted version.

## Conflict of Interest

The authors declare that the research was conducted in the absence of any commercial or financial relationships that could be construed as a potential conflict of interest.

## Publisher's Note

All claims expressed in this article are solely those of the authors and do not necessarily represent those of their affiliated organizations, or those of the publisher, the editors and the reviewers. Any product that may be evaluated in this article, or claim that may be made by its manufacturer, is not guaranteed or endorsed by the publisher.

## Abbreviations

AIH: autoimmune hepatitis
ALD: alcoholic liver disease
ANOVA: analysis of variance
AUC: area under curve
BMI: body mass index
E: emergency
ET: Eurotransplant
LT: liver transplantation
MELD: Model for End-Stage Liver Disease
O: outcome
U: utility
U_w_: weighted utility
HCC: hepatocellular carcinoma
NASH: non-alcoholic steatohepatitis
PBC: primary biliary cirrhosis
PSC: primary sclerosing cholangitis
ROC: receiver operator characteristic
SD: standard deviation.

## References

[1] Starzl TE Gordon RD Tzakis A Staschak S Fioravanti V Broznick B. Equitable allocation of extrarenal organs: with special reference to the liver. Transplant Proc. (1988) 20:131–8. 3278456 PMC2954650

[2] Aby ES Lake JR. Basic principles of liver allocation and development over the years. Curr Opin Organ Transplant. (2020) 25:99–103. 10.1097/MOT.0000000000000732 32073495

[3] Durand F. Development and outcomes of the French liver allocation system. Curr Opin Organ Transplant. (2020) 25:132–8. 10.1097/MOT.0000000000000749 32073486

[4] Tschuor C Ferrarese A Kuemmerli C Dutkowski P Burra P Clavien PA. Allocation of liver grafts worldwide - Is there a best system? J Hepatol. (2019) 71:707–18. 10.1016/j.jhep.2019.08.001 31199941

[5] Jost U Ringe B de Boer J Mühlbacher F Neuhaus P Otte JB. Preliminary experience with a new liver allocation system within Eurotransplant. Transplant Proc. (1993) 25:1547–9. 8442183

[6] Schlitt HJ Loss M Scherer MN Becker T Jauch KW Nashan B. [Current developments in liver transplantation in Germany: MELD-based organ allocation and incentives for transplant centres]. Z Gastroenterol. (2011) 49:30–8. 10.1055/s-0029-1245946 21225535

[7] Tacke F Kroy DC Barreiros AP Neumann UP. Liver transplantation in Germany. Liver Transpl. (2016) 22:1136–42. 10.1002/lt.24461 27082951

[8] Adam R Karam V Cailliez V O Grady JG Mirza D Cherqui D. 2018 Annual Report of the European Liver Transplant Registry (ELTR) - 50-year evolution of liver transplantation. Transplant Int. (2018) 31:1293–317. 10.1111/tri.13358 30259574

[9] Seehofer D Schöning W Neuhaus P. [Deceased donor liver transplantation]. Chirurg. (2013) 84:391–7. 10.1007/s00104-012-2413-8 23576123

[10] Kwong A Kim WR Lake JR Smith JM Schladt DP Skeans MA. OPTN/SRTR 2018 annual data report: liver. Am J Transplant. (2020) 20(Suppl. 1):193–299. 10.1111/ajt.15674 31898413

[11] NHS Blood and Transplant. Organ Donation and Transplantation Activity Report 2019/20. NHS.

[12] Blok JJ Braat AE Adam R Burroughs AK Putter H Kooreman NG. Validation of the donor risk index in orthotopic liver transplantation within the Eurotransplant region. Liver Transpl. (2012) 18:112–9. 10.1002/lt.22447 21987454

[13] Burton JR Jr Sonnenberg A Rosen HR. Retransplantation for recurrent hepatitis C in the MELD era: maximizing utility. Liver Transpl. (2004) 10:S59–64. 10.1002/lt.20259 15382221

[14] Molinari M Ayloo S Tsung A Jorgensen D Tevar A Rahman SH. Prediction of perioperative mortality of cadaveric liver transplant recipients during their evaluations. Transplantation. (2019) 103:e297–e307. 10.1097/TP.0000000000002810 31283673 PMC6756253

[15] Wunderlich H Brockmann JG Voigt R Rauchfuss F Pascher A Brose S. DTG procurement guidelines in heart beating donors. Transplant Int. (2011) 24:733–57. 10.1111/j.1432-2277.2011.01266.x 21668528

[16] Habib S Berk B Chang CC Demetris AJ Fontes P Dvorchik I. MELD and prediction of post-liver transplantation survival. Liver Transpl. (2006) 12:440–7. 10.1002/lt.20721 16498643

[17] Rana A Hardy MA Halazun KJ Woodland DC Ratner LE Samstein B. Survival outcomes following liver transplantation (SOFT) score: a novel method to predict patient survival following liver transplantation. Am J Transplant. (2008) 8:2537–46. 10.1111/j.1600-6143.2008.02400.x 18945283

[18] Dutkowski P Oberkofler CE Slankamenac K Puhan MA Schadde E Müllhaupt B. Are there better guidelines for allocation in liver transplantation? A novel score targeting justice and utility in the model for end-stage liver disease era. Ann Surg. (2011) 254:745–53; discussion 753. 10.1097/SLA.0b013e3182365081 22042468

[19] Wiesner R Edwards E Freeman R Harper A Kim R Kamath P. Model for end-stage liver disease (MELD) and allocation of donor livers. Gastroenterology. (2003) 124:91–6. 10.1053/gast.2003.50016 12512033

[20] Tsai MS Wang YC Wang HH Lee PH Jeng LB Kao CH. Pre-existing diabetes and risks of morbidity and mortality after liver transplantation: a nationwide database study in an Asian population. Eur J Intern Med. (2015) 26:433–8. 10.1016/j.ejim.2015.05.010 26048000

[21] Wang YJ Li JH Guan Y Xie QH Hao CM Wang ZX. Diabetes mellitus is a risk factor of acute kidney injury in liver transplantation patients. Hepatobiliary Pancreat Dis Int. (2021) 20:215–21. 10.1016/j.hbpd.2021.02.006 33752999

[22] Richtlinien zur Organtransplantation gem. Richtlinie gemäß § 16 Abs. 1 S. 1 Nrn. 2 u. 5 TPG für die Wartelistenführung und Organvermittlung zur Lebertransplantation. Deutsches Ärzteblatt (2019). p. 1–19.

[23] Merion RM Schaubel DE Dykstra DM Freeman RB Port FK Wolfe RA. The survival benefit of liver transplantation. Am J Transplant. (2005) 5:307–13. 10.1111/j.1600-6143.2004.00703.x 15643990

[24] Schaubel DE Guidinger MK Biggins SW Kalbfleisch JD Pomfret EA Sharma P. Survival benefit-based deceased-donor liver allocation. Am J Transplant. (2009) 9:970–81. 10.1111/j.1600-6143.2009.02571.x 19341419 PMC2895923

[25] Weismüller TJ Fikatas P Schmidt J Barreiros AP Otto G Beckebaum S. Multicentric evaluation of model for end-stage liver disease-based allocation and survival after liver transplantation in Germany–limitations of the ‘sickest first'-concept. Transplant Int. (2011) 24:91–9. 10.1111/j.1432-2277.2010.01161.x 20819196

[26] Feng S Goodrich NP Bragg-Gresham JL Dykstra DM Punch JD DebRoy MA. Characteristics associated with liver graft failure: the concept of a donor risk index. Am J Transplant. (2006) 6:783–90. 10.1111/j.1600-6143.2006.01242.x 16539636

[27] Braat AE Blok JJ Putter H Adam R Burroughs AK Rahmel AO. The Eurotransplant donor risk index in liver transplantation: ET-DRI. Am J Transplant. (2012) 12:2789–96. 10.1111/j.1600-6143.2012.04195.x 22823098

[28] Olthoff KM Kulik L Samstein B Kaminski M Abecassis M Emond J. Validation of a current definition of early allograft dysfunction in liver transplant recipients and analysis of risk factors. Liver Transpl. (2010) 16:943–9. 10.1002/lt.22091 20677285

